# An Assessment of Dementia Knowledge and Its Associated Factors Among Health College Students in Saudi Arabia

**DOI:** 10.7759/cureus.34578

**Published:** 2023-02-03

**Authors:** Sarah S Aldharman, Faisal T Alayed, Badr S Aljohani, Aliah M Aladwani, Meshal A Alyousef, Khalid M Hakami, Danah M Albalawi, Saud A Alnaaim

**Affiliations:** 1 College of Medicine, King Saud Bin Abdulaziz University for Health Sciences, Riyadh, SAU; 2 College of Medicine, Al-Imam Mohammed Ibn Saud Islamic University, Riyadh, SAU; 3 Department of Medicine, Alrayan Medical College, Madinah, SAU; 4 College of Medicine, Taif University, Taif, SAU; 5 College of Medicine, Al-Imam Mohammad Ibn Saud Islamic University, Riyadh, SAU; 6 College of Medicine, Jazan University, Jazan, SAU; 7 College of Medicine, University of Tabuk, Tabuk, SAU; 8 Department of Neurology, King Faisal University, Al-Ahsa, SAU

**Keywords:** saudi arabia, health students, associated factors, knowledge, dementia

## Abstract

Background

Dementia is a public health concern and the main cause of impairment and dependency among the elderly worldwide. It is characterized by a progressive decline in cognition, memory, and all domains of quality of life with preserving the level of consciousness. Accurate measurement of dementia knowledge among future health professionals is required to improve targeted educational initiatives and supportive care of patients. This study aimed to assess knowledge of dementia and its associated factors among health college students in Saudi Arabia.

Methods

A descriptive, cross-sectional study was conducted among health college students from various regions in Saudi Arabia. Data on sociodemographic characteristics and dementia knowledge were gathered using a standardized study questionnaire Dementia Knowledge Assessment Scale (DKAS) distributed on different social media platforms. Data analysis was carried out using IBM SPSS Statistics for Windows, Version 24.0 (IBM Corp., Armonk, NY, USA) statistical software. A *P*-value of <0.05 was considered significant.

Results

A total of 1,613 participants were included in the study. The mean age was 20.5 ± 2.5 years (range 18-25 years). The majority of them were males (64.9%), and females represented 35.1%. The mean knowledge score of the participants was 13.68 ± 3.18 (out of 25). According to DKAS subscales, we found that the respondents scored the highest in care considerations (4.17 ± 1.30) and the lowest in risks and health promotion (2.89 ± 1.96). Furthermore, we found that the participants with no previous dementia exposure had a significantly higher level of knowledge than those with previous dementia exposure. In addition, we found that both genders, aged 19, 21, 22, 23, 24, and 25 years; the geographic distribution of respondents; and previous dementia exposure significantly affected the DKAS score.

Conclusions

Our findings showed that health college students in Saudi Arabia had poor knowledge about dementia. Ongoing health education and comprehensive academic training are recommended to improve their knowledge and provide competent care for dementia patients.

## Introduction

Rapid demographic transitions due to increased life expectancy have resulted in a growing prevalence of the elderly population [1]. According to the World Health Organization (WHO), the number of people aged 60 and more years is predicted to rise over the next 15 years and more than double by 2050 [1]. Dementia is one of the major public health concerns associated with the elderly population. It can be distinguished by the following characteristics: progressive decline in cognitive ability, memory, or function that interferes with day-to-day activities, with intact consciousness [2]. Alzheimer’s disease is considered the most prevalent cause of dementia worldwide. Vascular disease, dementia with Lewy bodies, and frontotemporal dementia are some of the other prevalent causes of dementia [3]. Dementia might lead to a significant impact on social behavior, which makes life difficult not only for those populations but also for caregivers. Dementia is the seventh leading cause of death worldwide [4].

The overall prevalence of dementia is estimated to be about 55 million worldwide, with an increase in the incidence of nearly 10 million patients diagnosed every year based on recent data revealed by the WHO [4]. Furthermore, a study done in Saudi Arabia showed a high prevalence of dementia among elderly patients (16.6%) [5]. For future generations to be more equipped and involved in the care of dementia, dementia education is essential and should be included in the national health-related curriculum [6]. Numerous research conducted over several years has revealed that healthcare students lack proper training and knowledge on dementia [7-10].

This study aimed to assess the impact of a virtual dementia experience on medical and pharmacy students’ knowledge and attitudes toward individuals with dementia [11]. Medical and pharmacy students completed the dementia attitudes scale (DAS) pre-and postintervention. A total of 278 students (n  =  64 medical and n   = 214 pharmacies) were evaluated (n  =  80 intervention and n  =  198 control). The control group consisted of medical and pharmacy students who participated in the standard curriculum only, while the intervention group received a 1.5-hour multisensory, virtual simulation of light, sound, color, and visual content to experience the cognitive and perceptual difficulties faced by people with dementia. The study concluded that the intervention enhanced the DAS total score and that the intervention had a good impact on the knowledge and attitudes of medical and pharmacy students toward people with dementia [11].

Another study in Brazil evaluated students' knowledge and attitude toward dementia in the last year of medical college [12]. In regards to knowledge, the students scored a mean of 6.9, out of a scale of 0 to 14 points. For attitudes, the students believed that they can improve the patient's and caregiver's quality of life and that providing the diagnosis to the family is helpful [12].

A study conducted in Australia aimed to assess students' ability in medical school to recall the risk factors for dementia and cardiovascular disease (CVD) [13]. It showed that their knowledge of vascular risk factors for dementia was particularly insufficient. However, their general knowledge of dementia and Dementia Knowledge Assessment Scale (DKAS) scores were satisfactory [13]. A study done in Malaysia found that the overall dementia knowledge among ﬁnal year medical undergraduates from different public and private medical institutions was low [1]. In another study conducted on the general population, 32.5% of the participants knew about dementia, whereas 67.5% reveal no or little knowledge. Each gender, age, and education level significantly aﬀected the level of knowledge (*P* = 0.01, 0.04, and 0.02, respectively). There was a lack of knowledge among the general population about dementia, and they had a negative attitude toward it [14].

Moreover, another study was conducted in Kuwait to evaluate the knowledge of dementia among university students across diﬀerent campuses using DKAS. The results showed that the students in diﬀerent campuses had a signiﬁcant diﬀerence in the dementia total score as well as a signiﬁcant diﬀerence between all subscales of the DKAS. The mean total score of dementia knowledge among students on all campuses was 15.09 out of 25. The findings indicated that improved knowledge of dementia is required among students [15]. To the best of our knowledge, there is a gap of knowledge considering our topic in Saudi Arabia. Thereover, this study aimed to assess knowledge of dementia and determine its associated factors among health college students in Saudi Arabia.

## Materials and methods

This descriptive cross-sectional study was performed in Saudi Arabia between September 2022 and December 2022. The focus subjects were health college students from different regions of Saudi Arabia. The study was performed through a self-administered questionnaire delivered on various online platforms. All responses were gathered and transported to a Microsoft Excel file for processing information. Data were analyzed using IBM SPSS Statistics for Windows, Version 24.0 (IBM Corp., Armonk, NY, USA) statistical software.

Sample size estimation 

OpenEpi® version 3.0 (Centers for Disease Control and Prevention (CDC), Atlanta, GA, USA) software was employed to estimate the sample size. The representative sample size required was 385, with a margin error determined as 5%, a confidence level determined as 95%, and the population determined as approximately 20,000. We sought to get more than the estimated sample size to account for any exclusions. A nonprobability convenience sampling technique has been employed.

Inclusion and exclusion criteria

The study's eligibility criteria were any student from a health-related college including (medicine, pharmacy, dentistry, respiratory therapy, nursing, emergency medical services, clinical laboratory sciences, occupational therapy, radiology sciences, clinical nutrition, and speech therapy), from any region of Saudi Arabia, both genders, and aged ≥18 years. Participants who did not meet our inclusion criteria or who did not complete the questionnaire or who did not agree to participate were excluded.

Data collection instruments and procedures

We employed a self‐administered questionnaire, which was adapted from a previous study on comparable objectives [1]. The questionnaire contains two sections. The first section contains the participant’s demographic information including age, gender, region, medical school year, and previous dementia exposure. Previous dementia exposure was evaluated with the following items: previous exposure to formal dementia education (such as attending an educational session for health professionals or training course/workshop about dementia), if there was any immediate family member (parents, siblings, spouse, children, or anyone under your guardianship) diagnosed with dementia, and if there was any direct occupational/working experience in caring for dementia patients. The second section contains the DKAS. 

DKAS is a Likert scale that was originally designed as a 27-item knowledge questionnaire in the Wicking Dementia Research and Education Centre, University of Tasmania, Hobart, TAS, Australia, in 2015, with correct and incorrect statements about dementia [16]. It was updated in 2017 to a 25-item Likert scale, which was utilized in this study [17]. The DKAS was selected because of its good reliability [18,19]. The DKAS encompasses four subscales of dementia: causes and characteristics, communication and behavior, care considerations, and risks and health promotion. The DKAS research was conducted using the original English version since the study population was able to read and understand English.

The questionnaire has a total score of 50 points and is scored using the following Likert scale scoring system: score 2 points for an answer of *true* to a truthful statement or *false* to an untrue statement; score 1 point for an answer of *probably true* to a truthful statement or *probably false* to an untrue statement; and score 0 points for an answer of *true* or *probably true* to an untrue statement or *false* or *probably false* to a truthful statement or for an answer of *I don’t know*.

The questionnaire was distributed electronically using Google forms on different social media platforms such as WhatsApp, Twitter, and Telegram. All information was kept private and used for scientific research. Participation in this study was voluntary and optional, with informed consent provided to all participants on the first page before filling in the questionnaire. The ethical approval of the study was obtained before initiating the study. The ethical approval was obtained from the Research Ethics Committee at King Faisal University (Reference no. KFU-REC-2022-SEP-ETHICS178).

Statistical analysis

After distributing the questionnaire, it was checked for completeness and any missing information. The collected data was first entered into a Microsoft Excel file. Statistical analysis was conducted using SPSS Version 24.0 statistical software. The mean ± SD was used for continuous variables, while categorical variables were presented using frequencies and percentages. Categorical variables were compared using a chi-square test. A *P*-value of <0.05 was deemed significant.

## Results

Characteristics of the study participants

This study involved a total of 1,613 participants. About two-thirds of them were males (64.9%) and 35.1% were females. The average age of the study population was 20.5 ± 2.5 years (range 18-25 years), and about 40.2% were aged 18 years. According to the geographic distribution of respondents, we noted that almost half of them were from the central region (49.2%), followed by the western region (17.4%), eastern region (16.9%), southern region (11.3%), and northern region (only 5.1%). The characteristics of the study participants are shown in Table 1.

**Table 1:** Characteristics of the study participants (n = 1,613).

Variable	Categories	Frequency	Percentage (%)
Gender	Male	1,047	64.9
	Female	566	35.1
Age (years)	18	649	40.2
	19	64	4
	20	90	5.6
	21	163	10.1
	22	212	13.1
	23	212	13.1
	24	112	6.9
	25	111	6.9
Region	Northern	82	5.1
	Southern	183	11.3
	Central	794	49.2
	Eastern	273	16.9
	Western	281	17.4

**Figure 1:**
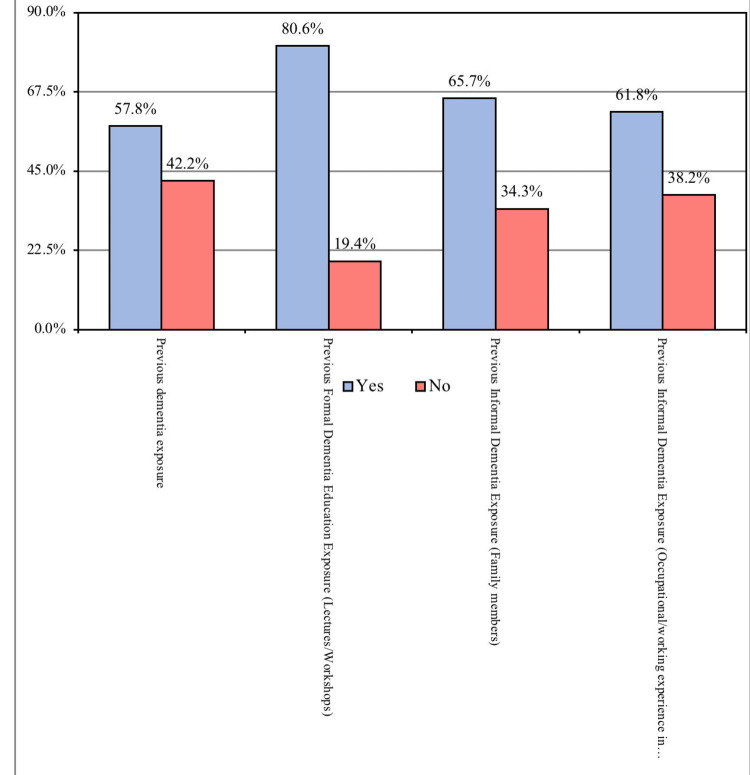
Previous dementia exposure.

Concerning previous dementia exposure, we observed that more than half of the participants (57.8%) admitted that they have experienced dementia exposure before, whereas 42.2% had not. Moreover, the majority of the respondents (80.6%) revealed that they had previous formal dementia education exposure through tools such as lectures and workshops. Most of the study population (65.7%) reported that they had informal dementia exposure previously by their family members, while 61.8% experienced this type of exposure in their occupational settings and in caring for dementia patients. Figure 1 illustrates the participants' previous dementia exposure. 

Total score and DKAS subscale scores in relation to previous dementia exposure

According to the DKAS scoring system, our results showed that the mean knowledge score of participants was 13.68 ± 3.18 (out of 25; Table 2). Furthermore, we found that the participants who had no previous dementia exposure had a significantly higher level of knowledge than the participants who had previous dementia exposure (knowledge score = 15.05 ± 3.33 and 12.69 ± 2.66, respectively; *P*-value < 0.001).

**Table 2:** Total score and DKAS subscale scores in relation to previous dementia exposure. DKAS, Dementia Knowledge Assessment Scale; SD, standard deviation

DKAS	No. of items (total score)	Mean ± SD	Previous dementia exposure (*n* = 933)	No previous dementia exposure (*n* = 680)	*P*-value
Total score	25 (25)	13.68 ± 3.18	12.69 ± 2.66	15.05 ± 3.33	<0.001
Subscale					
Causes and characteristics	7 (7)	3.72 ± 1.36	3.47 ± 1.16	4.07 ± 1.53	<0.001
Communication and behavior	6 (6)	2.90 ± 0.99	2.95 ± 0.85	2.84 ± 1.15	0.024
Care considerations	6 (6)	4.17 ± 1.30	3.86 ± 1.09	4.60 ± 1.45	<0.001
Risks and health promotion	6 (6)	2.89 ± 1.96	2.41 ± 2.18	3.55 ± 1.34	<0.001

When assessing DKAS subscales, we found that respondents scored the highest score in care considerations (4.17 ± 1.30) and the lowest score in risks and health promotion (2.89 ± 1.96). Regarding the causes and characteristics subscale, the mean score was 3.72 ± 1.36. Respondents with no previous dementia exposure scored higher than those with previous dementia exposure, and this difference was found to be statistically significant (*P*-value < 0.001). However, in the assessment of communication and behavior, respondents with previous dementia exposure revealed a higher level of knowledge than the participants who had no previous dementia exposure (*P*-value = 0.024). When we analyzed the care considerations knowledge subscale, participants with no previous exposure revealed a higher level of knowledge than those who had previous exposure (*P*-value < 0.001). Concerning risks and health promotion knowledge, participants with no previous dementia exposure scored higher than those with previous dementia exposure, and this difference was found to be statistically significant (*P*-value < 0.001).

Differences in DKAS scores between previous dementia exposure and demographic characteristics of the study participants

Our study revealed that there was a significant association between previous dementia exposure and both genders (*P*-value < 0.001). Males and females who had no previous exposure reported a higher level of knowledge than those who had previous exposure (scores = 14.61±3.28 and 15.32± 3.33, respectively).

Additionally, we found that the participants who had no previous dementia exposure and belonged to the age of 19, 21, 22, 23, 24, and 25 years were significantly associated with a higher level of knowledge about dementia (*P*-value = 0.012, 0.015, <0.001, 0.001, <0.001, and <0.001, respectively). The mean scores are demonstrated in Table 3.

**Table 3:** Differences in DKAS scores between previous dementia exposure and demographic characteristic of the study participants DKAS, Dementia Knowledge Assessment Scale; SD, standard deviation

Variable	Previous dementia exposure	*n* (%)	Mean ± SD	*P*-value
Gender				
Male	Yes	785 (75)	12.52 ± 2.42	<0.001
	No	262 (25)	14.61 ± 3.28	
Female	Yes	148 (26.1)	13.60 ± 3.52	<0.001
	No	418 (73.9)	15.32 ± 3.33	
Age (years)				
18	Yes	619 (95.4)	12.46 ± 2.22	0.070
	No	30 (4.6)	13.67 ± 3.47	
19	Yes	26 (40.6)	12.54 ± 2.93	0.012
	No	38 (59.4)	14.74 ± 3.58	
20	Yes	20 (22.2)	12.75 ± 2.47	0.197
	No	70 (77.8)	13.79 ± 3.30	
21	Yes	40 (24.5)	13.38 ± 2.95	0.015
	No	123 (75.5)	14.85 ± 3.37	
22	Yes	46 (21.7)	12.80 ± 3.44	<0.001
	No	166 (78.3)	15.16 ± 3.20	
23	Yes	68 (32.1)	14.15 ± 3.70	0.001
	No	144 (67.9)	15.86 ± 3.05	
24	Yes	51 (45.5)	12.73 ± 3.05	<0.001
	No	61 (54.5)	15.31 ± 3.51	
25	Yes	63 (56.8)	12.81 ± 3.46	<0.001
	No	48 (43.2)	15.40 ± 3.31	
Region				
Northern	Yes	23 (28)	12.09 ± 3.10	0.008
	No	59 (72)	14.22 ± 3.20	
Southern	Yes	68 (37.2)	12.57 ± 2.33	<0.001
	No	115 (62.8)	15.19 ± 3.11	
Central	Yes	658 (82.9)	12.50 ± 2.35	<0.001
	No	136 (17.1)	15.15 ± 3.24	
Eastern	Yes	104 (38.1)	13.45 ± 3.62	<0.001
	No	169 (61.9)	15.89 ± 3.37	
Western	Yes	80 (28.5)	13.53 ± 3.35	0.040
	No	201 (71.5)	14.44 ± 3.37	

Furthermore, the participants from northern, southern, central, western, and eastern regions who had no previous dementia exposure scored higher than those with previous dementia exposure from the same geographic regions. This difference was statistically significant (*P*-value = 0.008, <0.001, <0.001, <0.001, and 0.040, respectively). The mean scores are shown in Table 3.

Factors of previous dementia exposure affecting DKAS scores

Our findings reported that respondents with no previous formal dementia education exposure scored higher than those with previous formal dementia education exposure (lectures/workshops; mean score = 14.11 ± 3.37 and 13.58 ± 3.12, respectively; *P*-value < 0.001). Moreover, we noted that previous informal dementia exposure by family members did not significantly affect the DKAS score of our respondents (*P*-value < 0.192). Participants who had no previous informal exposure relatively achieved the highest knowledge score without any significant difference. Regarding previous informal dementia exposure in occupational/working experience in caring for dementia patients, we observed participants with no previous exposure scored higher than those with previous exposure (mean scores = 14.92 ± 3.45 and 12.92 ± 2.74, respectively). This difference was found to be significant (*P*-value < 0.001). Table 4 shows the factors of previous dementia exposure affecting DKAS scores.

**Table 4:** Factors of previous dementia exposure affecting DKAS scores. ^*^The factor that helps examine the relationship between the predictor and the outcome. DKAS, Dementia Knowledge Assessment Scale; SD, standard deviation; CI, confidence interval

Factors	Mean ± SD	*B*-value^*^	95% CI	*P*-value
Previous formal dementia education exposure (lectures/workshops)				
Yes	13.58 ± 3.12	0.758	0.336-1.179	<0.001
No	14.11 ± 3.37			
Previous informal dementia exposure (family members)				
Yes	13.28 ± 3.00	-0.246	-0.617 to 0.124	0.192
No	14.46 ± 3.36			
Previous informal dementia exposure (occupational/working experience in caring for dementia patients)				
Yes	12.92 ± 2.74	-2.149	-2.527 to -1.771	<0.001
No	14.92 ± 3.45			

The items of DKAS with the correct answers are presented in the Appendix.

## Discussion

This study aimed to investigate the knowledge level about dementia and its associated factors among health college students in Saudi Arabia. Dementia knowledge relates to knowledge and views about dementia recognition, management, and prevention [20]. In response to the increasing global frequency of this syndrome, the WHO urges more dementia awareness and education among those who provide care and treatment [21]. Health service employees [22], aged care workers [23], family caregivers [24], general practitioners [25], and students in health-related disciplines are among the target demographics for education [26]. There is clear evidence that dementia knowledge influences a person's health behavior.

Our results showed that the mean knowledge score of the participants was 13.68 ± 3.18 (out of 25). This score was lower than a previous study conducted in Malaysia, which revealed that overall dementia knowledge among participants with and without exposure was low, with an average score of 29.60 ± 6.97 and 28.22 ± 6.98, respectively [1]. Another study conducted in Hong Kong reported that the overall dementia knowledge score was 7.4 (SD = 3.7) out of 20 indicating a generally poor knowledge of dementia, which is in agreement with our results [7]. However, an earlier study in South Korea showed higher results - the average score and standard deviation for knowledge about dementia were 10.26 ± 1.24 out of 12 [27]. In this study, we found that participants who had no previous dementia exposure had a significantly higher level of knowledge than participants who had previous dementia exposure. One possible explanation for this conclusion is that the sources used by participants did not provide adequate knowledge about dementia in this study. However, respondents with prior dementia exposure demonstrated a better degree of knowledge than individuals who had no prior dementia exposure only in the communication and behavior subscale. This was consistent with another study, which found that people who had access to dementia material had much higher levels of dementia knowledge than others [27]. These disparities could be attributed to differences in target demographics and assessment technologies.

In the assessment of the communication and behavior subscale in our study, the participants with previous dementia exposure revealed a higher level of knowledge than the participants who had no previous dementia exposure (*P*-value = 0.024). On the other hand, in a South Korean study, a higher level of knowledge about dementia was associated with an increase in grade level (*P*  < 0.001), experience in education on dementia (*P* = 0.01), and previous experience in caring for people with dementia during clinical practice (*P* < 0.001) [27].

When we evaluated DKAS subscales, we found that respondents scored the highest in care considerations (4.17 ± 1.30) and lowest in risks and health promotion (2.89 ± 1.96). According to a Malaysian study, respondents rated the highest on the care consideration subscale (9.49 ± 2.37), which confirms our findings, and the lowest on the communication and conduct subscale (4.38 ± 2.39), which contradicts our findings [1].

Before the DKAS, dementia knowledge measures had been tested and developed with relatively small (e.g., *n =* 500) and narrowly defined populations (e.g., undergraduate health students), lacked generalizability, focused primarily on biomedical domains or specific types or stages of dementia (e.g., Alzheimer's disease), and had ceiling effects among educated respondents, simplistic response categories, and item ambiguity [28].

Furthermore, our findings revealed that participants with no prior dementia exposure and aged 19, 21, 22, 23, 24, or 25 years were substantially related to a greater level of dementia knowledge. Previous research found that younger age and self-reported dementia anxiety were substantially related to higher levels of dementia awareness [29]. Younger people may have more access to dementia-related information, particularly online, and they may experience fewer English language difficulties, allowing them to obtain bilingual material. Furthermore, we discovered that earlier informal dementia exposure by family members did not affect our respondents' DKAS scores. This was not corresponding to another study, which revealed that students who had family members with dementia had significantly higher levels of dementia knowledge than those without such relatives [27]. Another study reported that a positive family history of dementia was significantly associated with higher levels of dementia knowledge [29]. Our findings reported that higher knowledge of dementia was associated with no previous formal dementia education and without informal occupational/working experience in caring for dementia patients, which was inconsistent with another study [1].

The Alzheimer's Disease Knowledge Scale (ADKS) was utilized to measure the level of dementia knowledge. A study included a total of 321 undergraduate students who underwent the ADKS questionnaire. The study identified a moderate dementia knowledge base (mean score 23.51 out of 30) among health and social care students compared to our study, which showed poor knowledge by using the DKAS scale, which showed dementia knowledge of 13.68 ± 3.18 (out of 25) [30]. In comparison with another study, the overall mean score of students’ dementia knowledge evaluated by the ADKS was 19.49 ± 2.82 out of 30, and students’ attitudes toward dementia were 29.92 ± 3.35 out of 40 [8].

For the current investigation, some limitations must be highlighted. This study examines dementia knowledge primarily from a medical science standpoint; however, additional elements such as dementia beliefs and cultural values must be examined in future investigations. It's possible that some respondents guessed the correct answer because they did not want to admit that they did not know the correct answer. For these reasons, the study's findings should be interpreted with caution before making any conclusions about health and social care students' dementia knowledge and efficacy. The study was limited by being a questionnaire, which naturally carries the potential of interviewer, recollection, and response bias. Also, most participants in the study were males, which may affect the interpretations and comparisons of the data. However, this study is considered the first nationwide study to establish baseline dementia knowledge among the largest sampled study of health college undergraduates from various health and medical institutions of the kingdom of Saudi Arabia. We used an online survey platform that was significantly beneficial as the choices were antecedently made through compulsory choice-designed answers and finesse any misinformation entry via the participants.

The following recommendations are made in consideration of the results and conclusions of the study. Based on the ﬁndings, the overall level of the baseline knowledge of dementia and its associated factors among health college students in Saudi Arabia is low. However, it was revealed that gender, age, and education level were factors that had an impact on the level of knowledge. Thus, the following recommendations are hereby presented: the national medical curriculum should be revised and improved to include academic training as well as informal occupational/work experience on dementia to help in preparing future healthcare providers to recognize dementia and provide competent care among aging Saudis. Educators should consider whether the dementia curriculum is adequate for students to deal with the challenges that come with dementia caregiving. Finally, future researchers may conduct a similar study in a diﬀerent setting and compare the knowledge level between different colleges and educational years to discover new knowledge about dementia knowledge and its associated factors.

## Conclusions

Our results concluded that health college students in Saudi Arabia had poor knowledge about dementia. These findings have important implications for the establishment of specialized dementia teaching programs for Saudi health students. Dementia-related clinical training and educational curriculum are required to enhance the knowledge and abilities required of healthcare workers who have immediate contact with patients or the public through health and social care fields.

## Appendices

**Table 5:** The items of DKAS with correct answers. The 25-item DKAS was taken from [17]. DKAS: Dementia Knowledge Assessment Scale

Item #	Statement content	Domains/factor names
1	Most forms of dementia do not generally shorten a person’s life [False]	Causes and characteristics
2	Blood vessel disease (vascular dementia) is the most common form of dementia [False]	Causes and characteristics
3	People can recover from the most common forms of dementia [False]	Causes and characteristics
4	Dementia is a normal part of the ageing process [False]	Causes and characteristics
5	Dementia does not result from physical changes in the brain [False]	Causes and characteristics
6	Planning for end of life care is generally not necessary following a diagnosis of dementia [False]	Causes and characteristics
7	Alzheimer’s disease is the most common form of dementia [True]	Causes and characteristics
8	It is impossible to communicate with a person who has advanced dementia [False]	Communication and behaviour
9	A person experiencing advanced dementia will not generally respond to changes in their physical environment [False]	Communication and behaviour
10	It is important to correct a person with dementia when they are confused [False]	Communication and behaviour
11	People experiencing advanced dementia often communicate through body language [True]	Communication and behaviour
12	Uncharacteristic behaviours in a person experiencing dementia are generally a response to unmet needs [True]	Communication and behaviour
13	Medications are the most effective way of treating behavioural symptoms of dementia [False]	Communication and behaviour
14	People experiencing dementia do not generally have problems making decisions [False]	Care considerations
15	Movement is generally affected in the later stages of dementia [True]	Care considerations
16	Difficulty eating and drinking generally occurs in the later stages of dementia [True]	Care considerations
17	People with advanced dementia may have difficulty speaking [True]	Care considerations
18	People experiencing dementia often have difficulty learning new skills [True]	Care considerations
19	Daily care for a person with advanced dementia is effective when it focuses on providing comfort [True]	Care considerations
20	Having high blood pressure increases a person’s risk of developing dementia [True]	Risks and health promotion
21	Maintaining a healthy lifestyle does not reduce the risk of developing the most common forms of dementia [False]	Risks and health promotion
22	Symptoms of depression can be mistaken for symptoms of dementia [True]	Risks and health promotion
23	The sudden onset of cognitive problems is characteristic of common forms of dementia [False]	Risks and health promotion
24	Exercise is generally beneficial for people experiencing dementia [True]	Risks and health promotion
25	Early diagnosis of dementia does not generally improve quality of life for people experiencing the condition [False]	Risks and health promotion
